# Proton pump inhibitors induced fungal dysbiosis in patients with gastroesophageal reflux disease

**DOI:** 10.3389/fcimb.2023.1205348

**Published:** 2023-08-17

**Authors:** Yichao Shi, Jianfeng Li, Shuntian Cai, Hong Zhao, Huijun Zhao, Gang Sun, Yunsheng Yang

**Affiliations:** ^1^ Department of Gastroenterology, Aerospace Center Hospital, Peking University Aerospace School of Clinical Medicine, Beijing, China; ^2^ Department of Gastroenterology and Hepatology, The First Medical Center, Chinese PLA General Hospital, Beijing, China; ^3^ Department of Neurology, The First Medical Center, Chinese PLA General Hospital, Beijing, China; ^4^ National Clinical Research Center for Geriatric Diseases, Chinese PLA General Hospital, Beijing, China

**Keywords:** gastroesophageal reflux disease, proton pump inhibitor, fecal mycobiota, *Candida*, gastric mucosal mycobiota

## Abstract

Gut mycobiota inhabits human gastrointestinal lumen and plays a role in human health and disease. We investigated the influence of proton pump inhibitors (PPIs) on gastric mucosal and fecal mycobiota in patients with gastroesophageal reflux diseases (GERD) by using Internal Transcribed Spacer 1 sequencing. A total of 65 participants were included, consisting of the healthy control (HC) group, GERD patients who did not use PPIs (nt-GERD), and GERD patients who used PPIs, which were further divided into short-term (s-PPI) and long-term PPI user (l-PPI) groups based on the duration of PPI use. The alpha diversity and beta diversity of gastric mucosal mycobiota in GERD patients with PPI use were significantly different from HCs, but there were no differences between s-PPI and l-PPI groups. LEfSe analysis identified *Candida* at the genus level as a biomarker for the s-PPI group when compared to the nt-GERD group. Meanwhile, *Candida*, *Nothojafnea*, *Rhizodermea*, *Ambispora*, and *Saccharicola* were more abundant in the l-PPI group than in the nt-GERD group. Furthermore, colonization of *Candida* in gastric mucosa was significantly increased after PPI treatment. However, there was no significant difference in *Candida* colonization between patients with endoscopic esophageal mucosal breaks and those without. There were significant differences in the fecal mycobiota composition between HCs and GERD patients regardless whether or not they used PPI. As compared to nt-GERD patient samples, there was a high abundance of *Alternaria*, *Aspergillus*, *Mycenella*, *Exserohilum*, and *Clitopilus* in the s-PPI group. In addition, there was a significantly higher abundance of *Alternaria*, *Aspergillus*, *Podospora*, *Phallus*, and *Monographella* in the l-PPI group than nt-GERD patients. In conclusion, our study indicates that dysbiosis of mycobiota was presented in GERD patients in both gastric mucosal and fecal mycobiota. PPI treatment may increase the colonization of *Candida* in the gastric mucosa in GERD patients.

## 1 Introduction

Gastroesophageal reflux disease (GERD) is a common gastrointestinal (GI) disorder in all ages and both genders. The symptoms or complications of GERD are directly associated with the reflux of gastric contents into the esophagus. The gastroesophageal reflux could lead to reflux esophagitis with mucosal break, Barrett’s esophagus (BE), and even esophageal adenocarcinoma (EA). Approximately 70% of patients with persistent or repeated reflux symptoms require long-term acid suppressant therapy (Freston and Triadafilopoulos, 2004), mostly with proton pump inhibitors (PPIs). Although PPI has successfully inhibited acid secretion and relieved acid-related reflux symptoms, studies have suggested that long-term PPI use may result in several side effects, such as increased abundance of commensals in the upper GI tract, lowered abundance of gut commensals (Imhann et al., 2016; Jackson et al., 2016), increased risk of infections by enteric bacteria (Barkun et al., 2012; Wombwell et al., 2018; Moayyedi et al., 2019) such as *Clostridium difficile* infection (CDI) (Janarthanan et al., 2012), increased incidence of bone fractures (Jo et al., 2015), iron deficiency anemia (Imai et al., 2018), vitamin B12 deficiency (Lam et al., 2013), and pneumonia (Herzig et al., 2009; Alhazzani et al., 2018).

Some researchers have assumed that dysbiosis of microbiota may play a role in the pathogenesis of these diseases. The distal esophagus microbiome is altered in Barrett’s esophagus and reflux esophagitis (Snider et al., 2016). Recent research has shown that microbiome contributes to the development of Barret’s esophagus and gastroesophageal adenocarcinoma (Amir et al., 2014; Yang et al., 2014; Abnet et al., 2018). Approximately 20% of gut bacterial taxa were significantly altered in PPI users compared with those of non-users (Imhann et al., 2017), collectively referred to as “mycobiome” or “mycobiota”, as well as fungi (Khomeriki, 2014).

There is growing evidence that human microbes, including fungi (Huseyin et al., 2017), play an important role in diseases and health. Previous studies have shown that fungi are associated with aggravation of several human diseases, including inflammatory bowel disease (IBD), graft versus host disease, Hirschsprung-associated enterocolitis, colorectal cancer, and hepatitis B virus infections, which emphasize the need for further study of human mycobiota (Chen et al., 2011; Iliev et al., 2012; van der Velden et al., 2013; Frykman et al., 2015; Luan et al., 2015; Sokol et al., 2017). Mycobiota dysbiosis inhibited the healing of inflammatory lesions in animal models (Kumamoto, 2011), and inflammation promotes mycobiota dysbiosis of the GI tract (Kumamoto, 2011). Mycobiota dysbiosis is also considered to be closely related to the efficacy of IBD treatment. Patients with IBD flare had a significant increased abundance of *Candida albicans*. Moreover, *C. albicans* significantly reduced the efficacy of FMT in the treatment of CDI in an animal model and was related to poor efficiency of FMT in humans (Zuo et al., 2018). In these mouse models, antifungal therapy restored the efficacy of FMT. Dysbiosis of mycobiota was also indicated to be involved in the development of visceral hypersensitivity, which is closely related to refractory GERD symptoms (Botschuijver et al., 2017; Tack and Pandolfino, 2018). However, to the best of our knowledge, there is little research on the role of mycobiota in GERD. In this study, we used sequencing to detect fungi community in the feces and gastric mucosa of untreated and PPI-treated GERD patients, and found that both the gastric mucosal mycobiota and fecal mycobiota were significantly different from the healthy controls (HC), regardless of whether PPI treatment was used or not, and PPI treatment resulted in increased colonization of *Candida*.

## 2 Materials and methods

### 2.1 Patient and sample collection

The study was approved by the ethics committee of the Chinese PLA General Hospital and all subjects signed informed consent.

Samples from HCs and GERD patients undergoing routine diagnostic upper GI endoscopy at the Chinese PLA General Hospital were collected from June 2013 to July 2014 for further analysis. The participants were 18–75 years old and of Han ethnicity living in the north area of China. The GERD patients should present reflux symptoms with or without esophageal erosions evidenced by endoscopy. Patients treated with PPIs should take these drugs at prescribed doses (i.e., omeprazole, 40 mg/day). Healthy control subjects did not complain of reflux symptoms, had no *H. pylori* infection and abnormalities under endoscopy, and did not take PPIs and other drugs within 1 month before the study. Study exclusion criteria include the following: subjects with gastric cancer, peptic ulcer, Barrett’s esophagus, or esophageal adenocarcinoma; those with and a history of GI or hepatobiliary surgery; pregnant and lactating women; and those taking drugs that could disrupt the microbiota, such as antibiotics and probiotics, within 1 month before sample collection.

A total of 65 subjects participated in the trial and were classified into one of four phenotypes based on PPI use and duration: 12 healthy people (HC group, age 44.58 ± 11.22 years, female/male: 6/6), 21 patients who had not taken PPIs (untreated GERD patient group, nt-GERD group, age 53 ± 13.46 years, female/male: 9/12), 14 patients who had taken PPIs for 2 months to 1 year (average time of 6 months) (short-term PPI treatment group, s-PPI group, age 50.14 ± 14.39 years, female/male: 6/8), and 18 patients who had taken PPIs for more than 1 year to 10 years (average time of 2 years) (long-term PPI treatment group, l-PPI group, age 47.28 ± 10.64 years, female/male: 11/7). The demographic and clinical information is shown in **Table 1**. There was no statistical difference in age and sex composition among groups.

**Table 1:** Clinical characteristics of participants in the study.

Group	nt-GERD	s-PPI	l-PPI	HC
Number	21	14	18	12
Sex (female:male)	9/12	6/8	11/7	6/6
Age (mean ± SD)	53 ± 13.46	50.14 ± 14.39	47.28 ± 10.64	44.58 ± 1 1.22
Endoscopy				
LA-A	12	10	7	–
LA-B	6	1	4	–
LA-C	1	0	0	–
Nonerosive	2	3	7	–

LA, Los Angeles Classification.

The mean age and sex were not significantly different between HCs and GERD patients with or without PPI use (p > 0.05).

#### 2.1.1 Sample collection

Fecal specimens were stored in a sterile cryotube (Cryogenic Vial, Corning Incorporated) containing RNAlater at −80°C. The mucosal specimens were endoscopically taken from the gastric antrum and placed in a 1.5-ml cryotube. The cryotubes with samples were quickly transferred to a liquid nitrogen tank for temporary storage, and then transferred to a −80°C refrigerator for long-term storage.

### 2.2 Sequencing

#### 2.2.1 Extraction of genome DNA

Total genomic DNA was extracted from the samples using the CTAB method. The purity and concentration of DNA was detected by agarose gel electrophoresis. The appropriate amount of the sample was taken in a centrifuge tube and diluted to 1 ng/μl with sterile water.

#### 2.2.2 Amplicon generation

Primer: ITS (Internal Transcribed Space) 1: ITS1F–ITS2. Genes were amplified using the specific primer with the barcode.

All PCR reactions were carried out in a 30-μl reactor containing 15 μl of Phusion^®^ High-Fidelity PCR Master Mix (New England Biolabs), 0.2 μM of forward and reverse primers, and approximately 10 ng of template DNA. Thermal cycling consisted of initial denaturation at 98°C for 1 min, then denaturation at 98°C for 10 s, annealing at 50°C for 30 s, extension at 72°C for 60 s for 30 cycles, and finally 72°C for 5 min.

#### 2.2.3 PCR product quantification and qualification

The PCR product was mixed in an equal density ratio with 1× loading buffer (containing SYB green) and electrophoresed on 2% agarose gel. Samples with a bright main band between 400 and 450 bps were chosen for subsequent procedures.

#### 2.2.4 PCR product mixing and purification

The PCR products were mixed in an equinity ratio and were purified using a GeneJET Gel Extraction Kit (Thermo Scientific).

#### 2.2.5 Library preparation and sequencing

Sequencing libraries were constructed using the NEB Next^®^ Ultra™ DNA Library Prep Kit (Illumina) (NEB, USA) according to the manufacturer’s instruction, and index code was added. Library quality assessment was performed on a Qubit@ 2.0 fluorometer (Thermo Scientific) and an Agilent Bioanalyzer 2100 system. Finally, the library was sequenced on the Illumina Misses platform and generated paired-end reads of 250 bp.

### 2.3 Data analysis

#### 2.3.1 Paired-end read assemblies

Paired-end reads of the original DNA segments were merged using FLASH. Paired-end reads were allocated to samples based on the unique barcodes.

#### 2.3.2 Bioinformatic workflow

The UPARSE-OTU and UPARSE-OTUref algorithms were used for sequence analysis in the UPARSE software package.

Alpha diversity and beta diversity were analyzed using Internal Perl scripts. OTUs were clustered at 97% identify. A representative sequence was selected for each OTU, and the taxonomic information was annotated for each representative sequence using the RDP classifier. We normalized the OTU table based on the minimum depth from the OTU table summary from diversity analysis. Rarefaction curves and Specaccum curves were generated according to observed features. Observed feature, Shannon, Chao1, and Simpson index were used to reveal alpha diversity. Bray–Curtis distance, weighted UniFrac distance, and Aitchison distance were used for microbiome community dissimilarity. Principal coordinate analysis (PCoA) was performed to reduce the dimensionality of the original variables.

Linear discriminant analysis (LDA) effect size (LEfSe) algorithm was applied to discover distinctive taxonomic features characterizing the groups of samples. Only taxa with LDA scores greater than 2 at a *p*-value <0.08 were considered significantly enriched. LinDA analysis was also performed using the LinDA function of the Microbiome Stat v1.1 R package (Zhou et al., 2022) to detect differences in fungal component abundance between groups and linear regression on data transformed by central logarithmic ratio, returning *p*-values adjusted by multiple tests. We generated extended error bar (EEB) plots to show that some properties differed in GERD patients with some filter parameters (ratio of effect proportion ≥2, difference between proportion ≥1, and *p*-value = 0.05) by using the STAMP program (available at http://stamp-software.com/).

#### 2.3.3 Statistical analysis

The R package Stats was used to perform statistical analysis. Continuous variables are demonstrated as the median with interquartile range or mean ± SD. *p*-value <0.05 was considered significantly different. Wilcoxon rank sum test and one-way ANOVA were used to compare between groups. Analysis of similarities (ANOSIM) was performed to determine the differences of mycobiota in these groups. Biomarkers of different groups were quantitatively analyzed by LEfSe value 0.08 for factorial Kruskal–Wallis test. Welch’s *t*-test was used to identify the relative abundance difference of individual taxa between groups. The Kruskal–Wallis test was used to compare the relative abundance difference of specific taxa among groups.

## 3 Results

After data processing, 3,544,805 tags from all gastric mucosa were yielded with an average tags/sample of 57,174 ± 27,253. Overall, 99.5% of the tags were assigned to the taxonomic group. Clustering sequences were performed with a 97% similarity threshold, and yielded 2,091 OTUs.

From all fecal samples, 3,790,351 tags were yielded with an average tags/sample of 72,891± 56,113, and 97.9% of the tags were identified to the taxonomic groups. A total of 2,399 OTUs were clustered for all fecal samples.

The Specaccum curve and rarefaction curve of gastric mucosa mycobiota (**Supplementary Figures 1A–C**) and fecal mycobiota (**Supplementary Figures 1D–F**) were shown in supplementary figures.

### 3.1 Fungi genera in the gastric mucosa

**Figure 1:**
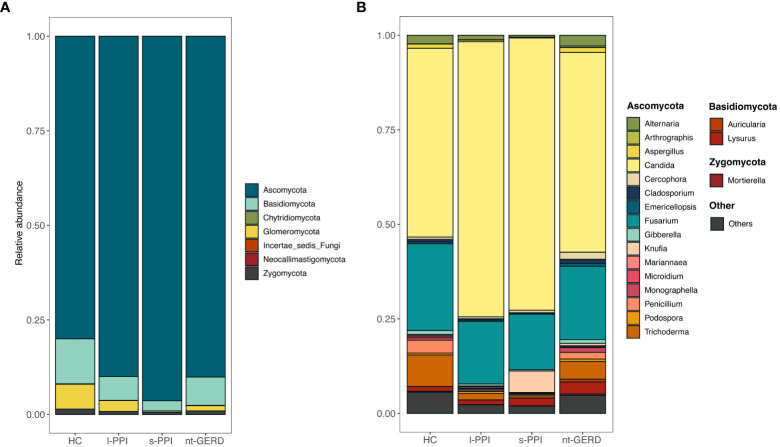
Stacked bar-plot representation of gastric mucosal mycobiota composition in nt-GERD, s-PPI, l-PPI, and HC with taxonomic features collapsed at the level of phyla and genera. **(A)** The composition of the fungi community at phylum levels among the groups. **(B)** The composition of the fungi community at genus levels among the groups. Only the top 20 taxa with the largest average relative abundance are listed.

The relative mycobiota community abundances at the phylum and genus levels were investigated from the gastric mucosal samples. The fungi of gastric mucosa were mainly composed of Ascomycota, Basidiomycota, Chytridiomycota, and Glomeromycota at the level of the phylum (**Figure 1A**). In the genus level, most abundant genera in the gastric mucosal mycobiota were *Candida*, *Fusarium*, *Trichoderma*, *Lysurus*, *Alternaria*, *Knufia*, *Penicillium*, *Cercophora*, *Aspergillus*, *Gibberella*, *Monographella*, and *Aspergillus* (**Figure 1B**).

**Figure 2:**
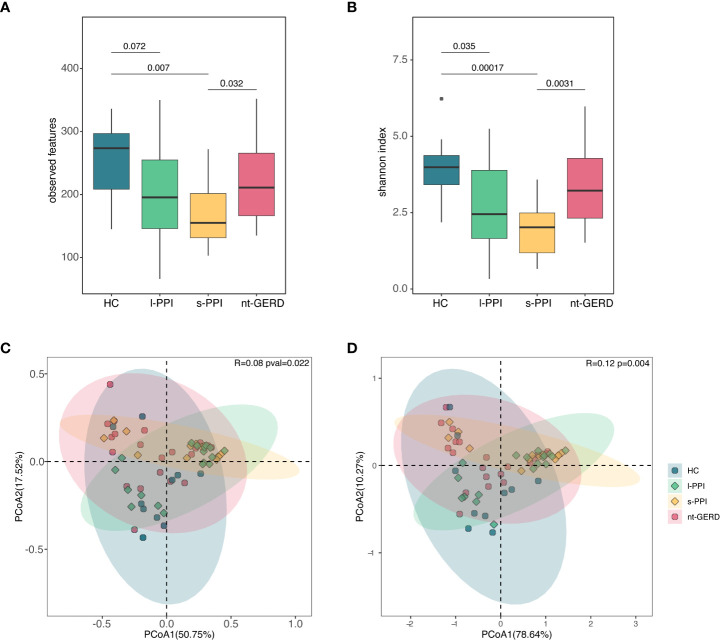
Comparison of alpha diversities, including observed features **(A)** and Shannon index **(B)** among the four groups at gastric mucosal mycobiota. Comparison of beta diversities, including Bray–Curtis dissimilarity index **(C)** and weighted UniFrac distance **(D)** among the four groups by the PCoA at gastric fungal communities.

The HC group and the l-PPI group had significantly greater fungi richness (observed features) than the s-PPI group in GERD patients (**Figure 2A**; **Supplementary Figure 2A**) and had higher abundance and evenness of the species present (Shannon and Simpson index) than the s-PPI group (**Figure 2B**; **Supplementary Figure 2B**). The Chao1 of gastric mucosal mycobiota among these four groups demonstrated no significant difference (**Supplementary Figure 2A**). The mucosal mycobiota observed features, Shannon index, and Simpson index of the s-PPI group were significantly lower than those of the nt-GERD group (**Figures 2A, B**; **Supplementary Figure 2B**). No significant difference was found in the alpha diversity between the s-PPI and l-PPI groups. In addition, no significant difference was found in the alpha diversity between the nt-GERD and l-PPI groups (**Figures 2A, B**; **Supplementary Figures 2A, B**).

We performed PCoA with Bray–Curtis distance matrix to investigate the microbial compositional differences among groups. Bray–Curtis distance revealed a statistically significant separation of the four groups (*R* = 0.08, *p* = 0.022), suggesting different mycobiota community structures (**Figure 2D**). There were differences in the fungal communities of the samples collected between the HC group and the s-PPI group (*p* = 0.015); meanwhile, there were also significant differences between the l-PPI group and the nt-GERD group (*p* = 0.030). This result was consistent with PCoA based on weighted UniFrac distances (*R* = 0.12, *p* = 0.004) (**Figure 2C**). However, Aitchison analysis found no significant difference among groups (*R* = 0.05, *p* = 0.075) (**Supplementary Figure 2C**).

**Figure 3:**
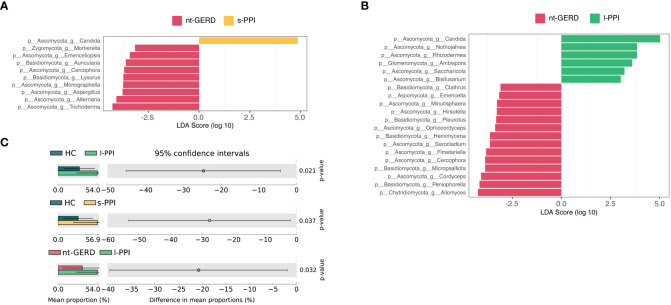
Linear discriminant analysis effect size (LEfSe) analysis of taxonomic features differentiating in gastric mucosal mycobiota between the nt-GERD group and the s-PPI group **(A)** and l-PPI group **(B)** (LDA values >2, *p* < 0.08) at the genus level. Differentially abundant taxa of each group are distinguished by different colors. *Candida* increased in patients treated with PPIs **(C)**. At the genus level, the abundance of *Candida* in patients treated with PPIs (s-PPI and l-PPI group) was significantly higher than in the nt-GERD group and healthy controls **(C)**.

LEfSe analysis (LDA >2, *p* < 0.08) were used to analyze the gastric mucosal fungi community structure in GERD patients with or without PPI use. In the genus level, we compared s-PPI or l-PPI with nt-GERD alone to investigate the potential taxonomic biomarkers. *Candida* was more abundant in the s-PPI group, while *Trichoderma*, *Alternaria*, *Aspergillus*, *Monographella*, and *Lysurus* were more abundant in the nt-GERD group (**Figure 3A**). *Candida*, *Nothojafnea*, *Rhizodermea*, *Ambispora*, and *Saccharicola* were more abundant in the l-PPI group, while *Allomyces*, *Peniophorella*, *Cordyceps*, *Micropsalliota*, and *Fimetariella* were more abundant in the nt-GERD group (**Figure 3B**). *Candida* increased in gastric mucosa of patients with short- or long-term PPI treatment. LinDA analysis results indicated that there was no significant difference in the relative abundance of fungi between GERD patients who used short-term or long-term PPI and the nt-GERD group (**Supplementary Figures 3A, B**).

### 3.2 *Candida* increased in gastric mucosa of patients with short- or long-term PPI treatment

We noticed that *Candida* was rich in the s-PPI and l-PPI groups compared to the nt-GERD group. Because *Candida* was one of the main gastric mucosal colonizing fungus genera and had important clinical significance, we compared the *Candida* relative abundance in the gastric mucosa of PPI-treated GERD patients, nt-GERD patients, and HCs using the Kruskal–Wallis test method. The relative abundance of *Candida* in the gastric mucosa of GERD patients treated with PPIs was significantly higher than that of nt-GERD patients and the HC group (**Figure 3C**). We further compared the relative abundance of *Candida* in patients with endoscopically visible esophageal mucosal lesions and patients without esophageal mucosal injury. The results showed that *Candida* in patients with endoscopically visible esophageal lesions was not significantly different from that in patients without visible lesions in gastric mucosa mycobiota (data not shown). Moreover, in GERD patients treated with PPI, the relative abundance of *Candida* in patients with endoscopically visible esophageal lesions was not significantly different from that in patients without visible lesions (data not shown). This result suggests that the mucosa after PPI treatment may provide a suitable environment for the growth of this mycobacteria, and its enrichment may be a biomarker of GERD patients after PPI use.

### 3.3 Fecal mycobiota

**Figure 4:**
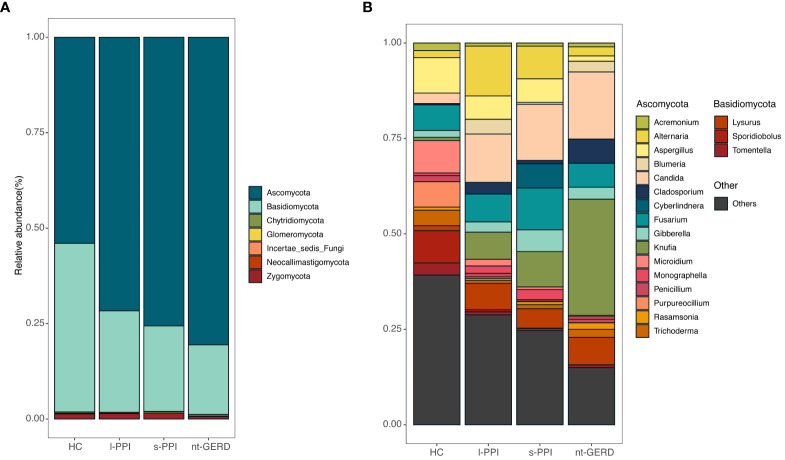
Stacked bar-plot representation of fecal mycobiota composition in nt-GERD, s-PPI, l-PPI, and HC with taxonomic features collapsed at the level of phyla and genera. **(A)** The composition of the fungi community at phylum levels among the groups. **(B)** The composition of the fungi community at genus levels among the groups. Only the top 20 taxa with the largest average relative abundance are listed.

The fecal samples of all the subjects were analyzed at the phylum and genus levels to obtain the taxonomic compositions. At the phylum level, the dominant fungi in the feces of all the subjects belonged to Ascomycota and Basidiomycota (**Figure 4A**). At the genus level, the mycobiota were mainly composed of *Cladosporium*, *Alternaria*, *Aspergillus*, *Penicillium*, *Rasamsonia*, *Knufia*, *Blumeria*, *Microidium*, *Candida*, *Cyberlindnera*, *Trichoderma*, and *Acremonium* (**Figure 4B**).

**Figure 5:**
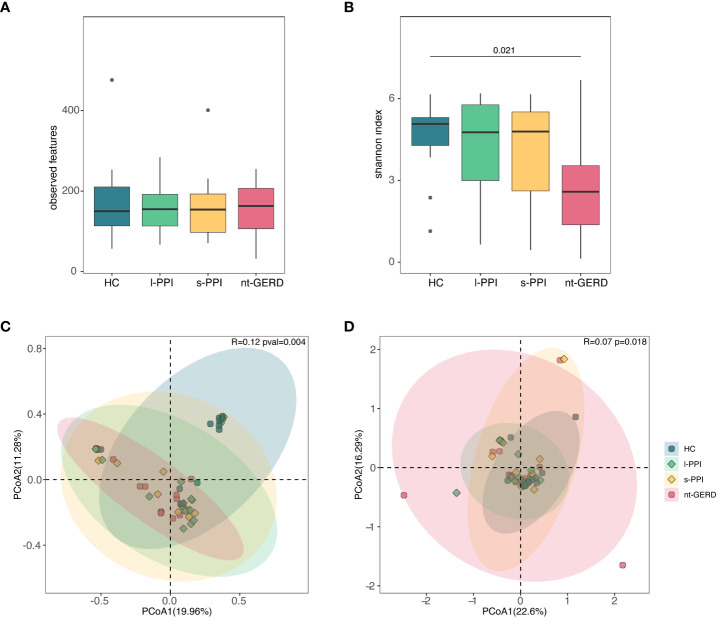
Comparison of alpha diversities, including observed features **(A)** and Shannon index **(B)** among the four groups at fecal mycobiota. Comparison of beta diversities, including Bray–Curtis dissimilarity index **(C)** and weighted UniFrac distance **(D)** among the four groups by the PCoA at fecal fungal communities.

There were no significant differences in the observed features and Chao1 index of the four groups (**Figure 5A**; **Supplementary Figure 2C**). The Shannon and Simpson index of the HC group was higher than those of the nt-GERD group (*p* < 0.05) (**Figure 5B**; **Supplementary Figure 2D**).

ANOSIM using PCoA based on Bray–Curtis showed that there was a significant difference in fecal fungal communities between the HC and the GERD groups, whether or not using PPIs (*R* = 0.12, *p* = 0.004) (**Figure 5C**), while there was no difference among the three groups of GERD patients. There were similar results when we performed weighted UniFrac analysis (*R* = 0.0718, *p* = 0.018) and Aitchison analysis (*R* = 0.06, *p* = 0.013) (**Figure 5D**; **Supplementary Figures 2G, H**).

**Figure 6:**
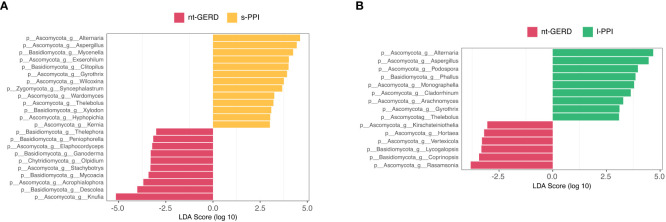
Linear discriminant analysis effect size (LEfSe) analysis of taxonomic features differentiating in fecal mycobiota between the nt-GERD group and the s-PPI group **(A)** and l-PPI group **(B)** (LDA values >2, *p* < 0.08) at the genus level. Differentially abundant taxa of each group are distinguished by different colors.

LEfSe analysis (LDA>2, *p* < 0.08) was used to analyze the fecal fungi community structure in GERD patients with or without PPI use. In the genus level, we compared s-PPI or l-PPI with nt-GERD alone to investigate the potential taxonomic biomarkers. *Alternaria*, *Aspergillus*, *Mycenella*, *Exserohilum*, and *Clitopilus* were more abundant in the s-PPI group, while *Knufia*, *Descolea*, *Acrophialophora*, *Mycoacia*, and *Stachybotrys* were more abundant in the nt-GERD group (**Figure 6A**). *Alternaria*, *Aspergillus*, *Podospora*, *Phallus*, and *Monographella* were more abundant in the l-PPI group, while *Rasamsonia*, *Coprinopsis*, *Lycogalopsis*, *Verteicola*, and *Hortaea* were more abundant in the nt-GERD group (**Figure 6B**). There was no obvious independent community between the s-PPI and nt-GERD group (**Supplementary Figure 3C**). LinDA analysis results showed that *Podospora* and *Aspergillus* were significantly different between the l-PPI and nt-GERD group (**Supplementary Figure 3D**).

## 4 Discussion

A greater proportion of Gram-negative anaerobes/microaerophiles (phyla Bacteroidetes, Proteobacteria, Fusobacteria, and Spirochaetes) were primarily associated with reflux esophagitis (RE, odds ratio, 15.4) and Barrett’s esophagus (BE) (odds ratio, 16.5) (Yang et al., 2009; Yang et al., 2014), which are usually caused by gastroesophageal reflux. Gastric microbiota in GERD is an understudied field, especially for the mycobiota due to the challenge in fungal annotation based on current genomic databases (Limon et al., 2017). Although the fungi only account for approximately 0.1% of the intestinal microbes, its individual volume is approximately 100 times that of bacteria, and many metabolic functions are uniquely found in fungi (Qin et al., 2010). In this study, we used ITS1 sequencing to investigate the fecal and gastric mucosal fungi alteration associated with GERD and PPI treatment. Feces and gastric mucosal fungi are mainly composed of Ascomycota and Basidiomycota in all the four groups, which is consistent with previous research in other diseases and healthy people (Sokol et al., 2017). In particular, our study showed that in both GERD patients and healthy controls, *Candida* were the dominant fungi of gastric mucosal fungi at the genus level. At the species level, *C. albicans* is the main dominant fungi in gastric mucosa. In this study, 96.9% of the subjects had *C. albicans* detected in the gastric mucosa with an average relative abundance of 43.59% (median 38.84%, 5%–95% percentiles 0.04%–91.23%) (data not shown). Only 1 of the 10 healthy controls did not detect *C. albicans*, and the detection rate was 90%, which was much higher than previous studies (Khomeriki, 2014), indicating that the colonization of *C. albicans* was underestimated. The relative abundance of *Candida* after PPI treatment was significantly higher than that of healthy controls and untreated GERD patients, but there was no difference between patients with short-term and long-term PPI treatment, indicating that PPI treatment can significantly increase the relative abundance of *Candida* in the stomach and colonization of *Candida* may not increase further after more than 2 months of PPI treatment. *Candida* may be responsible for the persistence of esophageal inflammation or symptoms as previous studies (Daniell, 2016) in HIV patients have shown that acid suppression could increase the incidence of *Candida* esophagitis. *C. albicans* abundance is associated with compromised FMT outcome in humans and a mouse model with CDI (Zuo et al., 2018). In addition, animal models show that host inflammation and colonization of *C. albicans* are synergistic factors (Jawhara et al., 2008). Therefore, we further compared the relative abundance of gastric mucosal *Candida* based on the presence or absence of endoscopic esophageal mucosal breaks. The results showed no significant difference in the relative abundance of gastric mucosal *Candida* between patients treated with PPIs with or without endoscopic esophageal mucosal breaks.

*C. albicans* was detected in the stool of 67.3% of the subjects in this study (data not shown), consistent with previous studies (Barcella et al., 2017). Some patients with GERD had a significant increase in *C. albicans* in the feces, but not found in healthy people (data not shown). *C. albicans* is one of the major pathogenic species and associated with polymicrobial infections (Allison et al., 2016). Limited by the small sample size and cross-sectional study type of this study, the effect of the increased *C. albicans* in feces was not analyzed.

Analysis of fungal alpha diversity and beta diversity suggested that the mycobiota of GERD patients was significantly different from healthy individuals, regardless of whether PPIs had been used. These results suggest that PPI treatment can effectively treat GERD, but it cannot restore the fungi flora imbalance, and may even cause further dysbiosis, because after PPI treatment, the mucosal fungal alpha diversity of GERD patients with PPI treatment was significantly reduced, while the alpha diversity of mucosal fungal of GERD patient without PPI treatment did not. In addition, two studies have suggested a significantly reduced alpha diversity of microbiota in PPI users (Imhann et al., 2016; Jackson et al., 2016). This may be due to several reasons. First, PPI treatment can significantly alter the pH value in the stomach, leading to fungal disorders through environmental changes. Secondly, PPIs can directly act on some microorganisms with proton pumps to affect the growth of fungi and bacteria (Vesper et al., 2009). In addition, PPIs can significantly alter gut microbiota (Takagi et al., 2018) and may further indirectly affect fungi through the interaction between bacteria and fungi (Sam et al., 2017). For example, the *Lactobacillus* within the stomach is effective at preventing colonization of *C. albicans* through displacement fungi from epithelium (Mason et al., 2012).

There is another possibility that most of the patients in this study still have symptoms and mucosal lesions after PPI treatment, which may have an impact on the results of the study if the disease state is the main factor affecting the flora. Prospective studies are worthwhile for a deeper understanding of the effects of PPIs and phenotype of disease on mycobiota.

In the past decade, acid suppression therapies, especially PPIs, have been proven effective in healing gastritis and esophagitis, concurrently with the increase in esophageal adenocarcinoma (EAC) incidence. Recently, a population-based cohort study that included all 796,492 adults indicated that maintenance PPI treatment was associated with standardized incidence ratios (SIRs) of esophageal adenocarcinoma (3.93, 95% CI 3.63–4.24) in the absence of other confounding factors (Brusselaers et al., 2018). Previous researchers hypothesize that the increased risk of carcinoma was due to a dysbiosis of the GI microbiome (Neto et al., 2016; Brusselaers et al., 2018). Moreover, dysbiosis of mycobiota caused by antifungals exacerbated the severity of DSS colitis and allergic airways (Wheeler et al., 2016). Gut mycobiota dysregulation in CDI patients is associated with FMT outcome, and it might be a possible causal relationship (Zuo et al., 2018). A previous study (Botschuijver et al., 2017) in humans and rats suggested that dysbiosis of intestinal fungi was associated with visceral hypersensitivity. Therapy with fungicide to hypersensitive rats restored the visceral hypersensitivity to normal levels, transfer of cecal mycobiomes from rats with visceral hypersensitivity established hypersensitivity to distension (Botschuijver et al., 2017). In our study, patients with GERD had significant mycobiota dysbiosis regardless of whether they are treated with PPIs, which may explain why most patients with PPI treatment for more than 2 months still had reflux symptoms and esophagitis, as underlying dysbiosis of microbiome, including mycobiome, might lead to host inflammation and visceral hypersensitivity.

Because the gut mycobiota was considered to be periodically fluctuating and less stable than bacterial microbiota (Dollive et al., 2013; Nash et al., 2017), more in-depth prospective research studies are needed to clarify the role of fungi in GERD. Caution should be exercised in interpreting the results of this study.

## 5 Conclusions

Gastric mucosal mycobiota and fecal mycobiota are dysregulated in patients with GERD. PPI treatment may not only fail to restore dysbiosis of mycobiome, but also cause an increase in colonization of *Candida* and cause further fungal dysbiosis in gastric mucosa and feces. Future longitudinal cohort studies or prospective studies will shed light on causality and deepen our understanding of the role of fungi in GERD. PPI treatments should be re-examined and should not be arbitrarily expanded.

## Data availability statement

The raw sequence data reported in this paper have been deposited in the Genome Sequence Archive (Genomics, Proteomics & Bioinformatics 2021) in National Genomics Data Center (Nucleic Acids Res 2022), China National Center for Bioinformation/Beijing Institute of Genomics, Chinese Academy of Sciences (GSA: CRA010685) that are publicly accessible at https://ngdc.cncb.ac.cn/gsa.

## Ethics statement

This study was undertaken with the approval of the Chinese PLA General Hospital Ethics Service Committee. All individuals enrolled in this study gave their informed consent.

## Author contributions

YS analyzed the data, drafted the manuscript, and funded the study; JL revised the manuscript; SC collected the samples; HZ, HJZ, and GS assisted analysis; YY designed and funded the study, and revised the manuscript. All authors contributed to the article and approved the submitted version.

## References

[B1] Abnet C. C. Arnold M. Wei W. Q. (2018). Epidemiology of esophageal squamous cell carcinoma. Gastroenterology 154, 360–373. doi: 10.1053/j.gastro.2017.08.023 28823862 PMC5836473

[B2] Alhazzani W. Alshamsi F. Belley-Cote E. Heels-Ansdell D. Brignardello-Petersen R. Alquraini M.. (2018). Efficacy and safety of stress ulcer prophylaxis in critically ill patients: a network meta-analysis of randomized trials. Intensive Care Med. 44, 1–11. doi: 10.1007/s00134-017-5005-8 29199388 PMC5770505

[B3] Allison D. L. Willems H. M. Jayatilake J. A. Bruno V. M. Peters B. M. Shirtliff M. E. (2016). Candida-bacteria interactions: their impact on human disease. Microbiol. Spectr. 4, 1–26. doi: 10.1128/9781555819286.ch5 27337476

[B4] Amir I. Konikoff F. M. Oppenheim M. Gophna U. Half E. E. (2014). Gastric microbiota is altered in oesophagitis and Barrett's oesophagus and further modified by proton pump inhibitors. Environ. Microbiol. 16, 2905–2914. doi: 10.1111/1462-2920.12285 24112768

[B5] Barcella L. Rogolino S. B. Barbaro A. P. (2017). The intestinal mycobiota: a year of observation about the incidence of yeast's isolation in fecal samples. Minerva Gastroenterol. Dietol 63, 85–91. doi: 10.23736/S1121-421X.17.02330-3 28150479

[B6] Barkun A. N. Bardou M. Pham C. Q. Martel M. (2012). Proton pump inhibitors vs. histamine 2 receptor antagonists for stress-related mucosal bleeding prophylaxis in critically ill patients: a meta-analysis. Am. J. Gastroenterol. 107, 507–520. doi: 10.1038/ajg.2011.474 22290403

[B7] Botschuijver S. Roeselers G. Levin E. Jonkers D. M. Welting O. Heinsbroek S. E. M.. (2017). Intestinal fungal dysbiosis is associated with visceral hypersensitivity in patients with irritable bowel syndrome and rats. Gastroenterology 153, 1026–1039. doi: 10.1053/j.gastro.2017.06.004 28624575

[B8] Brusselaers N. Engstrand L. Lagergren J. (2018). Maintenance proton pump inhibition therapy and risk of oesophageal cancer. Cancer Epidemiol. 53, 172–177. doi: 10.1016/j.canep.2018.02.004 29477057

[B9] Chen Y. Chen Z. Guo R. Chen N. Lu H. Huang S.. (2011). Correlation between gastrointestinal fungi and varying degrees of chronic hepatitis B virus infection. Diagn. Microbiol. Infect. Dis. 70, 492–498. doi: 10.1016/j.diagmicrobio.2010.04.005 20846815

[B10] Daniell H. W. (2016). Acid suppressing therapy as a risk factor for Candida esophagitis. Dis. Esophagus 29, 479–483. doi: 10.1111/dote.12354 25833302

[B11] Dollive S. Chen Y.-Y. Grunberg S. Bittinger K. Hoffmann C. Vandivier L.. (2013). Fungi of the murine gut: episodic variation and proliferation during antibiotic treatment. PloS One 8, e71806. doi: 10.1371/journal.pone.0071806 23977147 PMC3747063

[B12] Freston J. W. Triadafilopoulos G. (2004). Review article: approaches to the long-term management of adults with GERD-proton pump inhibitor therapy, laparoscopic fundoplication or endoscopic therapy? Aliment Pharmacol. Ther. 19 Suppl 1, 35–42. doi: 10.1111/j.0953-0673.2004.01837.x 14725577

[B13] Frykman P. K. Nordenskjöld A. Kawaguchi A. Hui T. T. Granström A. L. Cheng Z.. (2015). Characterization of bacterial and fungal microbiome in children with hirschsprung disease with and without a history of enterocolitis: A multicenter study. PloS One 10, e0124172. doi: 10.1371/journal.pone.0124172 25909773 PMC4409062

[B14] Herzig S. J. Howell M. D. Ngo L. H. Marcantonio E. R. (2009). Acid-suppressive medication use and the risk for hospital-acquired pneumonia. Jama 301, 2120–2128. doi: 10.1001/jama.2009.722 19470989

[B15] Huseyin C. E. O'toole P. W. Cotter P. D. Scanlan P. D. (2017). Forgotten fungi-the gut mycobiome in human health and disease. FEMS Microbiol. Rev. 41, 479–511. doi: 10.1093/femsre/fuw047 28430946

[B16] Iliev I. D. Funari V. A. Taylor K. D. Nguyen Q. Reyes C. N. Strom S. P.. (2012). Interactions between commensal fungi and the C-type lectin receptor Dectin-1 influence colitis. Science 336, 1314–1317. doi: 10.1126/science.1221789 22674328 PMC3432565

[B17] Imai R. Higuchi T. Morimoto M. Koyamada R. Okada S. (2018). Iron deficiency anemia due to the long-term use of a proton pump inhibitor. Intern. Med. 57, 899–901. doi: 10.2169/internalmedicine.9554-17 29151538 PMC5891535

[B18] Imhann F. Bonder M. J. Vich Vila A. Fu J. Mujagic Z. Vork L.. (2016). Proton pump inhibitors affect the gut microbiome. Gut 65, 740–748. doi: 10.1136/gutjnl-2015-310376 26657899 PMC4853569

[B19] Imhann F. Vich Vila A. Bonder M. J. Lopez Manosalva A. G. Koonen D. P. Y. Fu J.. (2017). The influence of proton pump inhibitors and other commonly used medication on the gut microbiota. Gut Microbes 8, 351–358. doi: 10.1080/19490976.2017.1284732 28118083 PMC5570416

[B20] Jackson M. A. Goodrich J. K. Maxan M. E. Freedberg D. E. Abrams J. A. Poole A. C.. (2016). Proton pump inhibitors alter the composition of the gut microbiota. Gut 65, 749–756. doi: 10.1136/gutjnl-2015-310861 26719299 PMC4853574

[B21] Janarthanan S. Ditah I. Adler D. G. Ehrinpreis M. N. (2012). Clostridium difficile-associated diarrhea and proton pump inhibitor therapy: a meta-analysis. Am. J. Gastroenterol. 107, 1001–1010. doi: 10.1038/ajg.2012.179 22710578

[B22] Jawhara S. Thuru X. Standaert-Vitse A. Jouault T. Mordon S. Sendid B.. (2008). Colonization of mice by Candida albicans is promoted by chemically induced colitis and augments inflammatory responses through galectin-3. J. Infect. Dis. 197, 972–980. doi: 10.1086/528990 18419533

[B23] Jo Y. Park E. Ahn S. B. Jo Y. K. Son B. Kim S. H.. (2015). A proton pump inhibitor's effect on bone metabolism mediated by osteoclast action in old age: A prospective randomized study. Gut Liver 9, 607–614. doi: 10.5009/gnl14135 25473078 PMC4562777

[B24] Khomeriki S. (2014). Standard therapeutic regimens in H. pylori infection leads to activation of transitory fungal flora in gastric mucus. Eksp Klin Gastroenterol. 105, 16–20. 25518495

[B25] Kumamoto C. A. (2011). Inflammation and gastrointestinal Candida colonization. Curr. Opin. Microbiol. 14, 386–391. doi: 10.1016/j.mib.2011.07.015 21802979 PMC3163673

[B26] Lam J. R. Schneider J. L. Zhao W. Corley D. A. (2013). Proton pump inhibitor and histamine 2 receptor antagonist use and vitamin B12 deficiency. Jama 310, 2435–2442. doi: 10.1001/jama.2013.280490 24327038

[B27] Limon J. J. Skalski J. H. Underhill D. M. (2017). Commensal fungi in health and disease. Cell Host Microbe 22, 156–165. doi: 10.1016/j.chom.2017.07.002 28799901 PMC5573128

[B28] Luan C. Xie L. Yang X. Miao H. Lv N. Zhang R.. (2015). Dysbiosis of fungal microbiota in the intestinal mucosa of patients with colorectal adenomas. Sci. Rep. 5, 7980. doi: 10.1038/srep07980 25613490 PMC4648387

[B29] Mason K. L. Erb Downward J. R. Falkowski N. R. Young V. B. Kao J. Y. Huffnagle G. B. (2012). Interplay between the gastric bacterial microbiota and Candida albicans during postantibiotic recolonization and gastritis. Infect. Immun. 80, 150–158. doi: 10.1128/IAI.05162-11 21986629 PMC3255670

[B30] Moayyedi P. Eikelboom J. W. Bosch J. Connolly S. J. Dyal L. Shestakovska O.. (2019). Safety of proton pump inhibitors based on a large, multi-year, randomized trial of patients receiving rivaroxaban or aspirin. Gastroenterology 157, 682–691.e682. doi: 10.1053/j.gastro.2019.05.056 31152740

[B31] Nash A. K. Auchtung T. A. Wong M. C. Smith D. P. Gesell J. R. Ross M. C.. (2017). The gut mycobiome of the Human Microbiome Project healthy cohort. Microbiome 5, 153. doi: 10.1186/s40168-017-0373-4 29178920 PMC5702186

[B32] Neto A. G. Whitaker A. Pei Z. (2016). Microbiome and potential targets for chemoprevention of esophageal adenocarcinoma. Semin. Oncol. 43, 86–96. doi: 10.1053/j.seminoncol.2015.09.005 26970127 PMC4789168

[B33] Qin J. Li R. Raes J. Arumugam M. Burgdorf K. S. Manichanh C.. (2010). A human gut microbial gene catalogue established by metagenomic sequencing. Nature 464, 59–65. doi: 10.1038/nature08821 20203603 PMC3779803

[B34] Sam Q. H. Chang M. W. Chai L. Y. (2017). The fungal mycobiome and its interaction with gut bacteria in the host. Int. J. Mol. Sci. 18, 1–11. doi: 10.3390/ijms18020330 PMC5343866 28165395

[B35] Snider E. J. Freedberg D. E. Abrams J. A. (2016). Potential role of the microbiome in barrett's esophagus and esophageal adenocarcinoma. Dig Dis. Sci. 61, 2217–2225. doi: 10.1007/s10620-016-4155-9 27068172 PMC4945493

[B36] Sokol H. Leducq V. Aschard H. Pham H. P. Jegou S. Landman C.. (2017). Fungal microbiota dysbiosis in IBD. Gut 66, 1039–1048. doi: 10.1136/gutjnl-2015-310746 26843508 PMC5532459

[B37] Tack J. Pandolfino J. E. (2018). Pathophysiology of gastroesophageal reflux disease. Gastroenterology 154, 277–288. doi: 10.1053/j.gastro.2017.09.047 29037470

[B38] Takagi T. Naito Y. Inoue R. Kashiwagi S. Uchiyama K. Mizushima K.. (2018). The influence of long-term use of proton pump inhibitors on the gut microbiota: an age-sex-matched case-control study. J. Clin. Biochem. Nutr. 62, 100–105. doi: 10.3164/jcbn.17-78 29371761 PMC5773837

[B39] van der Velden W. J. Netea M. G. De Haan A. F. Huls G. A. Donnelly J. P. Blijlevens N. M. (2013). Role of the mycobiome in human acute graft-versus-host disease. Biol. Blood Marrow Transplant. 19, 329–332. doi: 10.1016/j.bbmt.2012.11.008 23160005

[B40] Vesper B. J. Jawdi A. Altman K. W. Haines G. K. 3rd Tao L. Radosevich J. A. (2009). The effect of proton pump inhibitors on the human microbiota. Curr. Drug Metab. 10, 84–89. doi: 10.2174/138920009787048392 19149516

[B41] Wheeler M. L. Limon J. J. Bar A. S. Leal C. A. Gargus M. Tang J.. (2016). Immunological consequences of intestinal fungal dysbiosis. Cell Host Microbe 19, 865–873. doi: 10.1016/j.chom.2016.05.003 27237365 PMC4900921

[B42] Wombwell E. Chittum M. E. Leeser K. R. (2018). Inpatient proton pump inhibitor administration and hospital-acquired clostridium difficile infection: evidence and possible mechanism. Am. J. Med. 131, 244–249. doi: 10.1016/j.amjmed.2017.10.034 29122635

[B43] Yang L. Chaudhary N. Baghdadi J. Pei Z. (2014). Microbiome in reflux disorders and esophageal adenocarcinoma. Cancer J. 20, 207–210. doi: 10.1097/PPO.0000000000000044 24855009 PMC4120752

[B44] Yang L. Lu X. Nossa C. W. Francois F. Peek R. M. Pei Z. (2009). Inflammation and intestinal metaplasia of the distal esophagus are associated with alterations in the microbiome. Gastroenterology 137, 588–597. doi: 10.1053/j.gastro.2009.04.046 19394334 PMC2963147

[B45] Zhou H. He K. Chen J. Zhang X. (2022). LinDA: linear models for differential abundance analysis of microbiome compositional data. Genome Biol. 23, 95. doi: 10.1186/s13059-022-02655-5 35421994 PMC9012043

[B46] Zuo T. Wong S. H. Cheung C. P. Lam K. Lui R. Cheung K.. (2018). Gut fungal dysbiosis correlates with reduced efficacy of fecal microbiota transplantation in Clostridium difficile infection. Nat. Commun. 9, 3663. doi: 10.1038/s41467-018-06103-6 30202057 PMC6131390

