# Supplementary figures and images for: Proton pump inhibitors induced fungal dysbiosis in patients with gastroesophageal reflux disease
Source: Front Cell Infect Microbiol. 2023 Aug 17;13:1205348. doi: 10.3389/fcimb.2023.1205348 (PMC10469693; doi:10.3389/fcimb.2023.1205348)

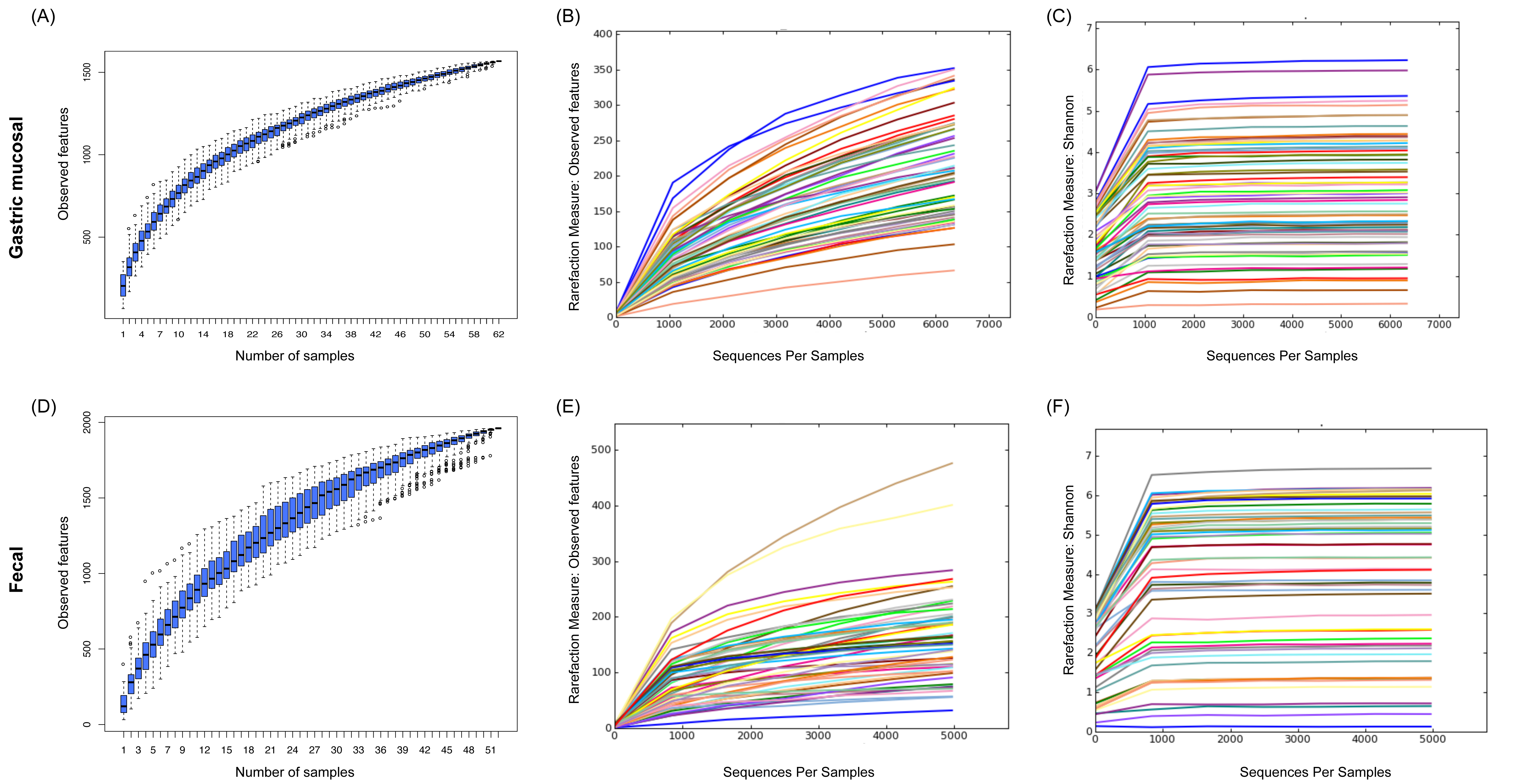

Supplement: Supplementary file 1 [file Image_1.jpeg]

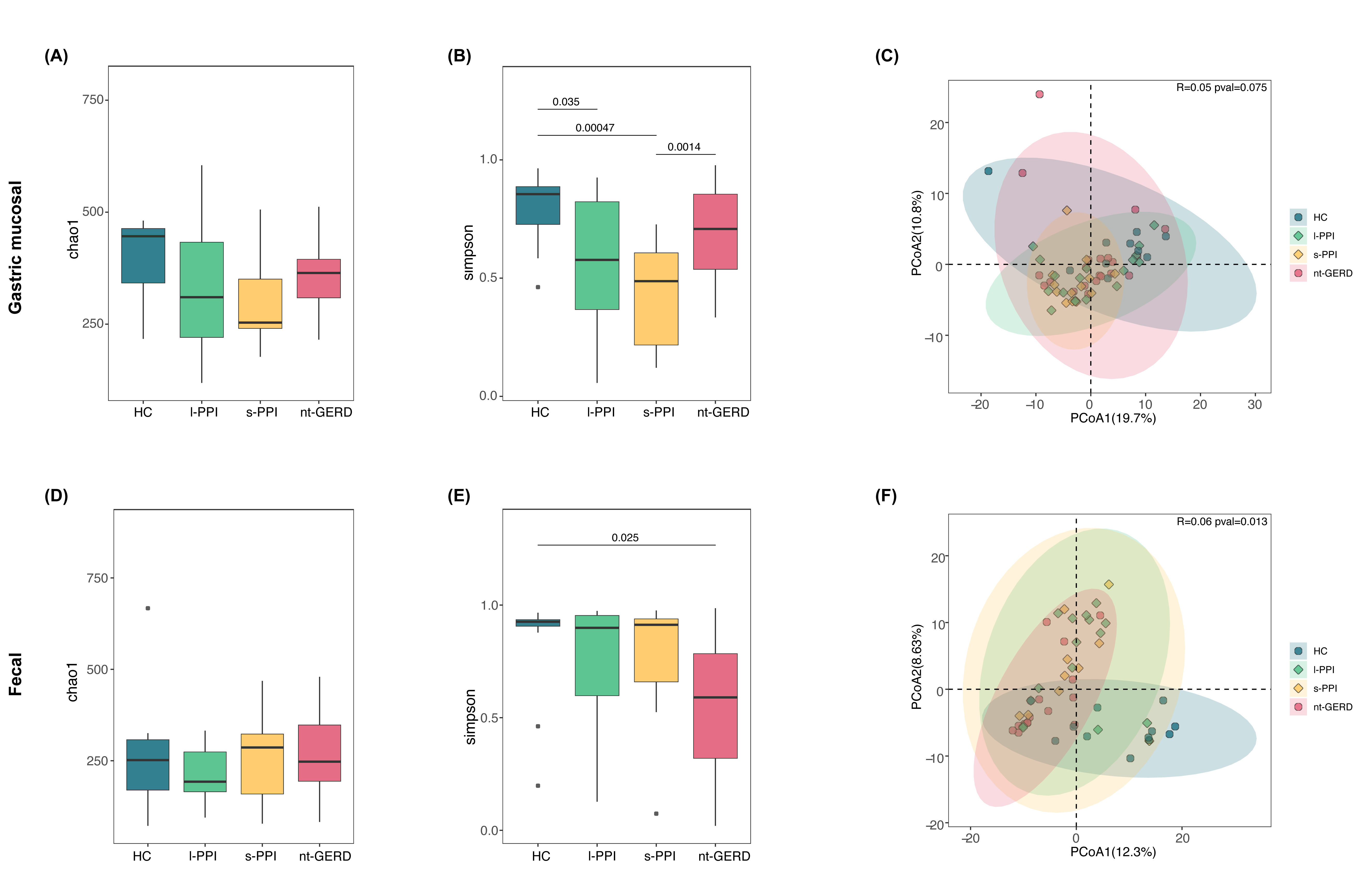

Supplement: Supplementary file 2 [file Image_2.jpeg]

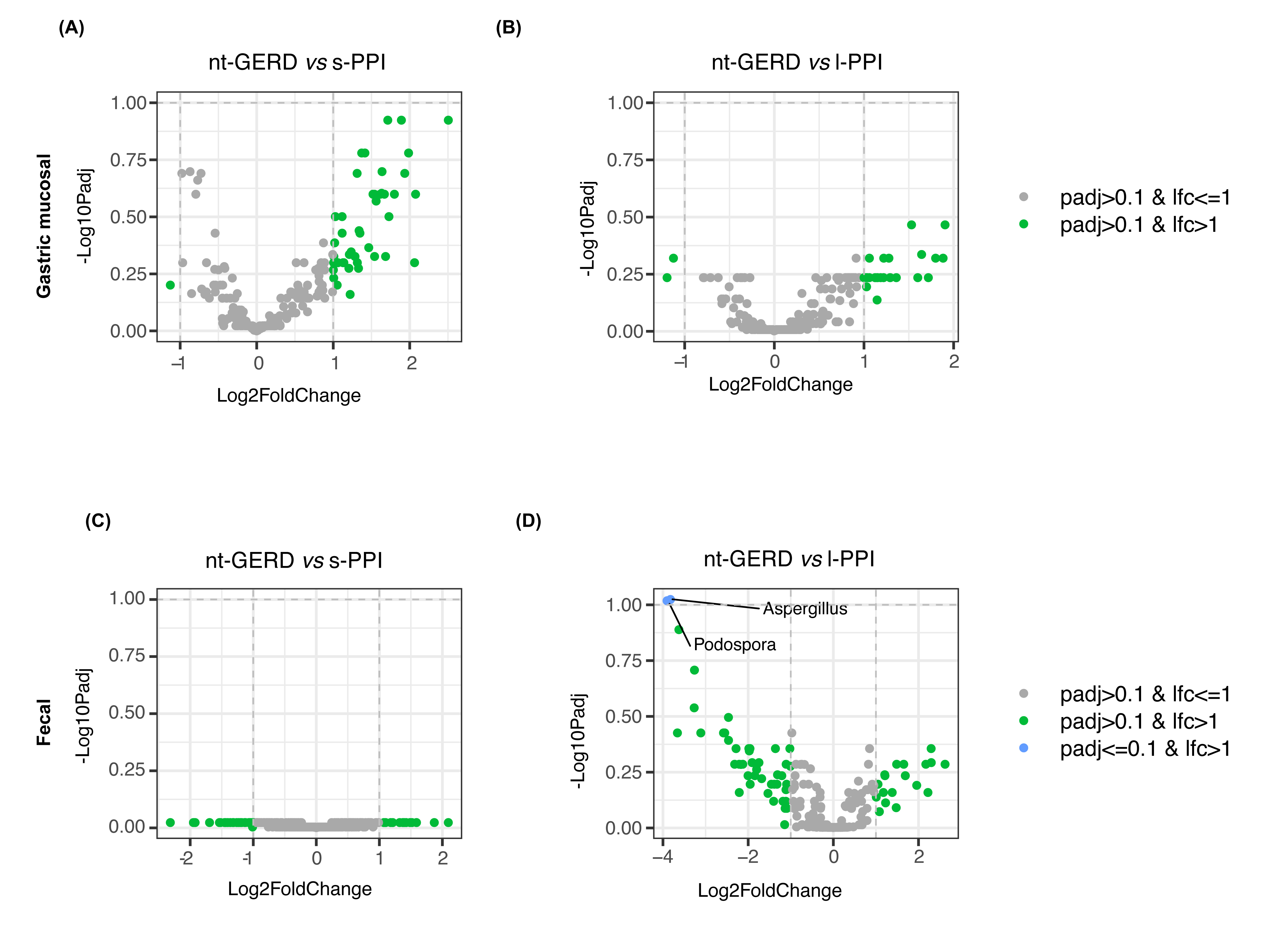

Supplement: Supplementary file 3 [file Image_3.jpeg]
