# Supplementary material for: Proton pump inhibitors induced fungal dysbiosis in patients with gastroesophageal reflux disease
Source: Front Cell Infect Microbiol. 2023 Aug 17;13:1205348. doi: 10.3389/fcimb.2023.1205348 (PMC10469693; doi:10.3389/fcimb.2023.1205348)
Supplement: Supplementary file 4 [file Table_1.docx]

Proton Pump Inhibitors induced Fungal Dysbiosisin Patients with Gastroesophageal Reflux Disease

Yichao Shi^1,2#^, Jianfeng Li^2#^, Shuntian Cai^2^, Hong Zhao^3^, Huijun Zhao^2^, Gang Sun^2^, Yunsheng Yang^2,4*^

^1^Department of Gastroenterology, Aerospace Center Hospital, Peking University Aerospace School of Clinical Medicine, Beijing 100049，China

^2^Department of Gastroenterology and Hepatology, the First Medical Center, Chinese PLA General Hospital, Beijing 100853, China

^3^ Department of Neurology, the First Medical Center, Chinese PLA General Hospital, Beijing 100853, China

^4^ National Clinical Research Center for Geriatric Diseases, Chinese PLA General Hospital, Beijing, China

^#^Equal contribution.

*** Correspondence:**Yunsheng Yang
sunnyddc@plagh.org

Supplementary figure 1-3

Supplementary Figure 1. Specaccum, rarefaction curve and rank abundance curve of gastric mucosa mycobiota (A-C) and fecal mycobiota (E-F).

Supplementary Figure 2. Comparison of alpha diversities, including Chao1 (A) and Simpson index (B) among the four groups at gastric mucosal mycobiota. Comparison of beta diversities, using Aitchison analysis to detect differences in gastric fungal communities among the four groups (C).

Supplementary Figure 3. The volcano plots for the two datasets (gastric mucosal, fecal) respectively. Volcano plot of differential taxa at FDR level of 0.1 for the mycobiota of gastric mucosal between s-PPI or l-PPI and nt-GERD (A, B), and also in fecal dataset (C, D). The taxa in black are detected by LinDA. Taxa in blue are the significantly difference genus between l-PPI and nt-GERD group.
